# ﻿*Vicialiupanshanensis* (Fabaceae), a new plant species from northwestern China

**DOI:** 10.3897/phytokeys.259.154664

**Published:** 2025-07-04

**Authors:** Bo Yang, Jun Yang, Siyu Wei, Hongmei Zhang, Xiaowei Li

**Affiliations:** 1 College of Forestry and Prataculture, Ningxia University, Yinchuan 750021, China Ningxia University Yinchuan China; 2 Ningxia Grassland and Animal Husbandry Engineering Technology Research Center, Ningxia University, Yinchuan 750021, China Ningxia University Yinchuan China; 3 Key Laboratory for Model Innovation in Forage Production Efficiency, Ministry of Agriculture and Rural Affairs, Ningxia University, Yinchuan 750021, China Ningxia University Yinchuan China; 4 Key Laboratory of Land Degradation and Ecological Restoration in Northwest China, Ningxia University, Yinchuan 750021, China Ningxia University Yinchuan China

**Keywords:** Leguminosae, new taxon, taxonomy, *Vicialiupanshanensis*

## Abstract

A new legume species, *Vicialiupanshanensis*, discovered in the southern Ningxia Hui Autonomous Region and central Gansu Province of northwestern China, is described and illustrated. The distinguishing characteristics of this herbaceous plant include its semi-sagittate stipules and the presence of 7–9 pairs of leaflets in its leaves. The racemose inflorescence, which is approximately equal in length to the leaf, features rhombic bractlets (5–7 mm long) covered with short, soft hairs. The secund flowers, arranged along one side of the inflorescence, are slightly drooping, approximately 1 cm in length. The corolla displays a shift in colour from pale greenish-creamy-white to yellow-brown, dark yellow, or dark orange. In accordance with the criteria established by the IUCN Red List, the species *Vicialiupanshanensis* is evaluated as being of Vulnerable status (VU).

## ﻿Introduction

The genus *Vicia* comprises leguminous herbs that are annual, biennial or perennial in nature. These plants frequently exhibit climbing, trailing or creeping growth habits and comprise 150–210 species found in Asia, Europe, and North America, the majority being native to the Mediterranean region (Cacan et al. 2016; Han et al. 2021). *Vicia* has also been observed to have colonised Hawaii in the Pacific and the middle Atlantic archipelagos of the Canaries, Madeira and Azores. Ball (1968) divided the genus into four sections: *Vicia*, *Faba*, *Ervum*, and *Cracca*, while Kupicha (1976) subsequently divided it into two subgenera, *Vicilla* and *Vicia*. Flora Reipublicae Popularis Sinicae (FRPS) records that there are 43 species and 5 varieties of *Vicia* in China, and classifies them into seven sections: *Cassubicae*, *Cracca*, *Ervum*, *Faba*, *Lenticula*, *Oroboidea*, and *Vicia*. The Flora of China (FOC) is an authoritative compendium that documents 40 species of *Vicia* in China (13 endemic and 3 introduced) (Bao and Turland. 2010). As of the beginning of 2025, there are 45 species, 5 subspecies, 14 varieties, and 9 forms of wild pea plants (http://www.sp2000.org.cn/). These plants are widely distributed across all provinces and regions of the country, reflecting their extensive distribution and adaptability. However, these species are comparatively more abundant in the northwestern, northern, and southwestern parts of China.

Research indicates that the genus *Vicia* is located in the Vicioid clade and is a polyphyletic group, with distinguishing features including square stems, inwardly curved and flattened hollow styles, and longitudinal rows of hairs on the inner side of the style (Schaefer et al. 2012). A previous systematic study of members in the genus *Vicia*, based on nuclear ribosomal ITS data and style morphology, demonstrated that both lateral compression of the style and dorsal clustering of trichomes, are more recently evolved features, whereas dorsoventral compression of the style and uniform pubescence are more primitive characteristics (Choi et al. 2006). It is estimated that more than 40 species of *Vicia* are cultivated due to their economic benefits (El-Bok et al. 2014). Some of these cultivated and domesticated species, such as *V.sativa*, *V.villosa*, *V.costata*, *V.bungei*, and *V.faba*, are now extensively cultivated in numerous countries worldwide (Yi et al. 2020; Ahmed and Sattar 2024; Ren et al. 2024). Recent studies have demonstrated the significant anti-tumour, anti-oxidative and anti-inflammatory properties of compounds isolated from *V.bungei* (Yang et al. 2023; Le et al. 2024).

In 2024, during the course of extensive botanical surveys in the Liupan Mountains, a *Vicia*-like plant was encountered in Laolongtan and the Mts. Daxueshan, in Jingyuan County, Ningxia Hui Autonomous Region and in Zhuanglang County, Gansu Province, This plant featured a corolla that was greenish-white at first, gradually turning yellow-brown, dark yellow or dark orange, and leaves with 7–9 pairs of lanceolate to elliptic leaflets. Comparison with relevant literature and specimens revealed similarities to *V.taipaica* and *V.mingyueshanensis*, yet distinct morphological differences (Table 1) indicate that it is an undescribed species, which is described herein as new.

**Table 1.** Detailed comparison of *Vicialiupanshanensis* and its two morphologically-similar conspecific species.

Characters	*V.liupanshanensis*	*V.taipaica*	*V.mingyueshanensis*
Stem height (cm)	50–150	60–100	50–180
Plant indumentum	Covered with fine soft hairs or nearly glabrous	Totally glabrous	Totally glabrous
Leaf length (cm), tendril excluded	8–15	8–12	8–15
Leaflet pairs per leaf	7–9	4–6	3–5
Leaflet shape	Oblong-lanceolate or elliptical	Elliptic to ovate-oblong	Elliptic to ovate-oblong
Leaflet size (cm)	1.7–2.5 × 0.6–0.9	1.3–5.0 × 0.6–1.5	2.3–3.8 × 0.7–1.5
Leaflet texture	Papery	Papery	Papery
Stipules	Semi-sagittate	Attached at the lower part, with the upper part being semi-ovate and the tip having a linear-lanceolate shape	Semi-hastate or lanceolate
Raceme (number of flowers)	10–15	5–15	10–20
Bractlet shape	5–7 mm long, rhombic, covered with short soft hairs	Absent	0.2–0.3 × 0.1 cm, subulate
Corolla color	Corolla pale greenish-white initially, gradually turning yellow-brown, dark yellow or dark orange	Yellow or brown-yellow	Light yellow or dull orange
Calyx shape	Oblique campanulate, hairy, with 5 teeth	Shortly and unequally toothed	Obliquely campanulate, 5 lateral teeth acute, some calyces are cleft
Standard	Ovate, about 1 cm long, with a broad claw, the apex concave, the sides constricted in the middle	Narrowly obovate-oblong, constricted at middle, ca. 13 mm, apex retuse	Apex retuse, 1.3–1.4 × 0.4–0.5 cm
Wing	Nearly equal (subequal) in length to the standard	10 mm	Nearly equal (subequal) in length to the standard
Keel	Slightly shorter than standard and wing	10 mm	Nearly equal (subequal) in length to the standard
Legume (mm)	Oblong, 20–30 × 4–5	Oblong-rhomboid, 20–30 × 5	Falcate, 30–35 × 3
Seed color	Brown with black spots	Brown with black spots	Brown-green
Number of seeds per pod	3–5	2–5	4–6

## ﻿Materials and methods

The specimens of this novel *Vicia* species were collected from forest edges of Daxueshan, in the Jingyuan County, Ningxia Hui Autonomous Region (106.3284 E, 35.3809N; 1972.91 m a.s.l.). These plant specimens have been stored in the Herbarium of the Department of Grassland Science at Ningxia University. We conducted extensive surveys in the Liupanshan region, and we collected 28 specimens of this species from Migangshan, Daxueshan, and Erlonghe in Jingyuan County, and 5 specimens from Zhuanglang County and Longde County near Jingyuan County.

We brought specimen plants from the above regions to the laboratory. Once there, some plants were measured for key plant characteristics; others were used to take colour photographs and draw illustrations, and the Flora of China and reports of new species of *Vicia* spp. in the last decade were consulted. We found that it is most similar to *Viciamingyueshanensis* and *Viciataipaica*, but there were obvious differences. Based on the measured empirical data, Adobe Illustrator 2024 software was used to add scale bars to each color photo. The species was then compared to a morphologically similar conspecific. Finally, its conservation status was then assessed according to the International Union for Conservation of Nature (IUCN) Red List criteria and corresponding guidelines (IUCN 2024), to evaluate the protected status of this new species, here named *Vicialiupanshanensis*. The type specimens and some specimens will be sent to the Herbarium of the Institute of Botany, Chinese Academy of Sciences (PE), while the remaining specimens are currently preserved at Ningxia University.

## ﻿Taxonomy

### ﻿ Vicia liupanshanensis

Taxon classificationPlantaeFabalesFabaceae

Xiao Wei Li & Bo Yang sp. nov.

A97F3C37-B5DC-537F-81C8-584B801B9713

urn:lsid:ipni.org:names:77364920-1

#### Description.

**Figure 1.**
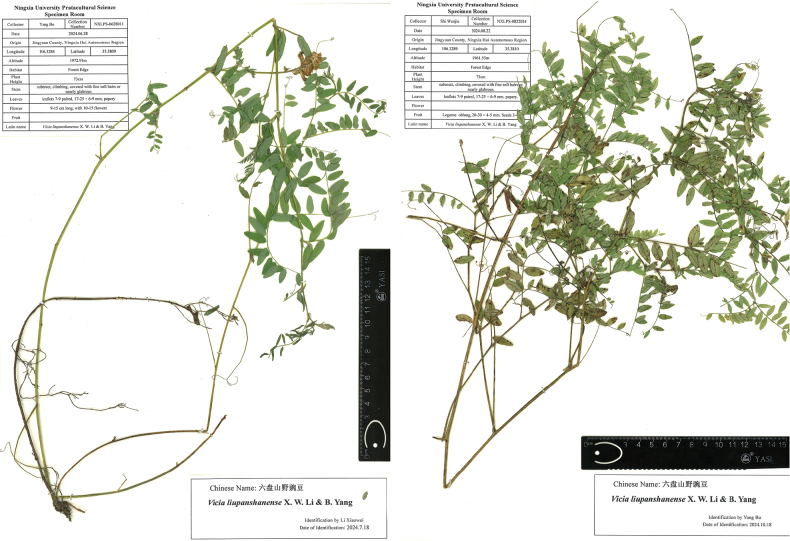
Photographs of two collected *Vicialiupanshanensis* specimens.

Herb perennial, 50–150 cm tall, stem erect, climbing, covered with fine soft hairs or nearly glabrous. Leaves even-pinnate, 8–15 cm; branched tendrils presented at apex with 3 or 4 branches; stipules semi-sagittate, 8–10 mm; leaflets 7–9 pairs, alternate, oblong-lanceolate or elliptical, 17–25 × 6–9 mm, papery, apex obtuse or acuminate, distinctly mucronate, base rounded or broadly cuneate, upper surface sparsely covered with short and soft hairs, lower surface covered with short hairs with veins densely hairy; veins prominent and raised on lower surface, petiolule ca. 1 mm. Inflorescence a raceme, about the same length as leaves, 8–15 cm long, with–15 flowers; bracts 5–7 mm × 4–5 mm, rhombic, covered with short and soft hairs. Flowers secund, slightly drooping, ca. 1 cm in length, pedicel ca. 1 mm; calyx obliquely campanulate, hairy; lobes 5, lanceolate, 1–3 mm long; corolla pale greenish-white at first, gradually turning yellow-brown, dark yellow or dark orange; standard ovate, ca. 8–10 mm × 4–5 mm, with a broad claw at base, concave at apex, margins constricted in the middle; wings nearly equal in length to the standard, obliquely ovate, auriculated, with a claw 4–5 mm at base; keels ca. 7–9 mm long, apex blunt, shortly auriculated. Ovary linear, glabrous 4–5 ovulued; the style hairy towards upper part; stigma capitate. Pods oblong, 20–30 × 4–5 mm. Seeds 3–5 × 3.5–4 mm, oblate-spheroid, brown, with black speckles. Flowering and fruiting June–August. Figs 1, 3, 4.

#### Type.

Jingyuan of Ningxia, forest edge (35.3809, 106.3284; 1972.91 m a.s.l.), June 28^th^ 2024, *Yang Bo NXLPS-0628011* (holotype: PE).

#### Etymology.

The species epithet derives from the name of the mountain range (Liupanshan) where this species was discovered.

#### Vernacular name.

The Chinese name is ‘六盘山野豌豆’ (Liù Pán Shān Yě Wān Dòu)

#### Distribution and habitat.

**Figure 2.**
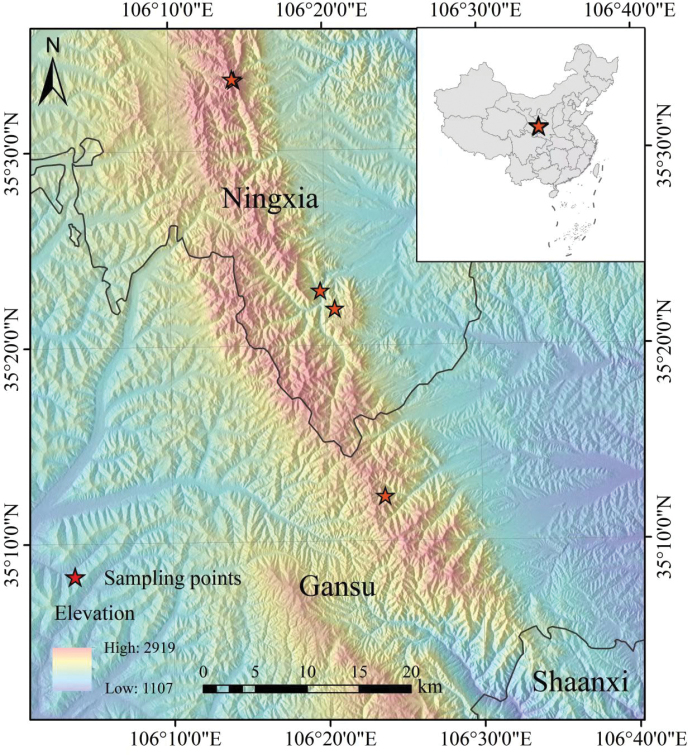
Sampling points where the *Vicialiupanshanensis* specimens were collected in China.

*Vicialiupanshanensis* occurs only at elevations of 1900–2300 m, in Laolongtan, Daxue Mountains, and Migang Mountains in Jingyuan Countyand Longde County, Ningxia Hui Autonomous Region and Zhuanglang County, Gansu Province, in China. It is generally found in alpine scrubs and hillside grasslands. (Fig. 2).

**Figure 3.**
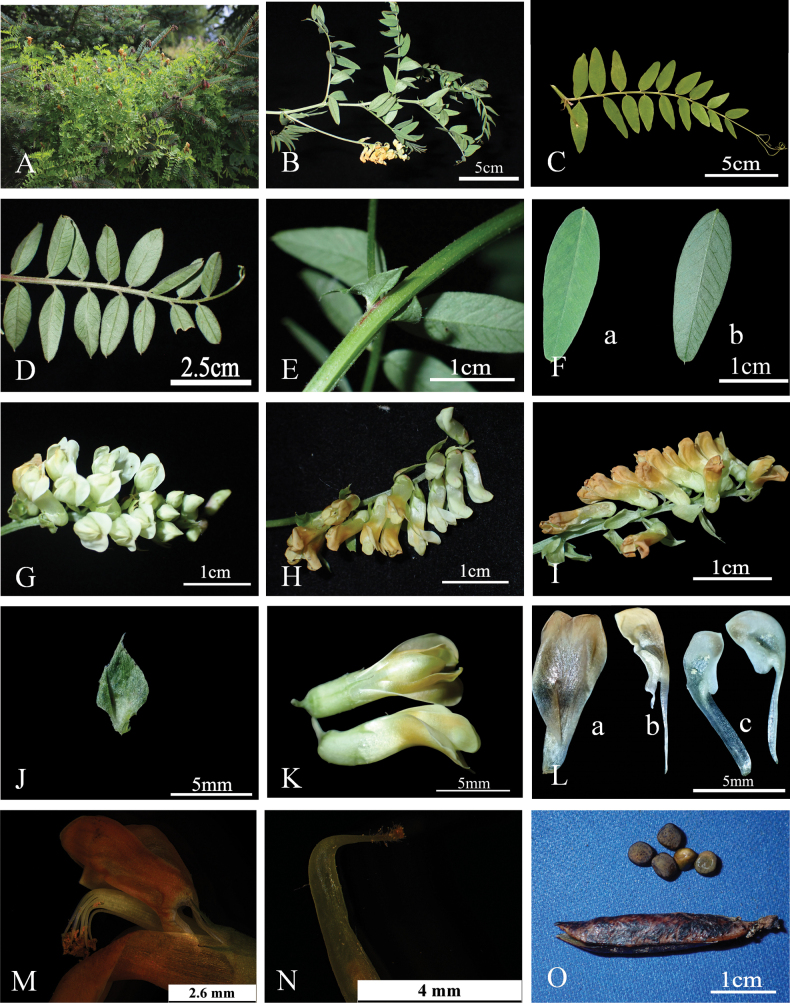
Photographs of *Vicialiupanshanensis* (Voucher specimen: NXLPS-0628011 and NXLPS-0822024). **A.** Life form and habit; **B.** Upper portion of a *V.liupanshanensis* individual; **C.** Leaf front; **D**. Leaf back; **E.** Leaves paripinnate; **Fa.** Leaflets front; **Fb.** Leaflets back; **G–I.** Inflorescence; **J.** Bractlet; **La.** Standard; **Lb.** Wing; **Lc.** Keels; **M.** Stamen; **N.** Stigma; **O.** Pod and seeds.

**Figure 4.**
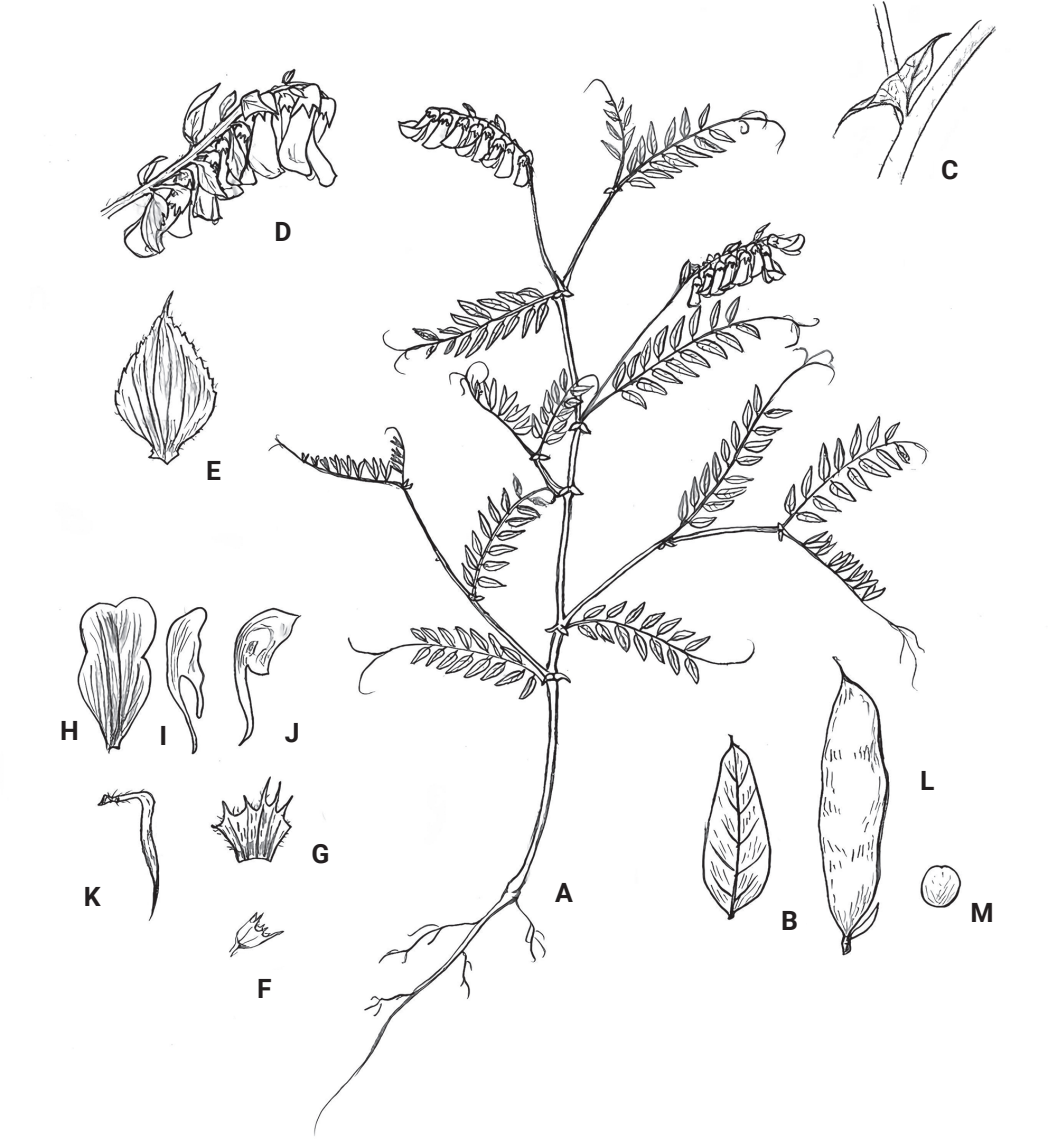
Drawings of *Vicialiupanshanensis* parts (Voucher specimen: NXLPS-0628011 and NXLPS-0822024). **A.** Whole plant; **B.** Leaflet; **C.** Leaves paripinnate; **D.** Inflorescence; **E.** Bractlet; **F, G.** Calyx; **H.** Standard; **I.** Wing; **J.** Keel; **K.** Stigma; **L.** Pod; and **M.** Seed.

#### Conservation assessment.

According to our preliminary conservation assessment, *Vicialiupanshanensis* is predominantly found in the Liupanshan National Nature Reserve within the Ningxia Hui Autonomous Region. Only four populations have been identified (so far), the rest are sporadic, whose total estimated number of mature individuals does not exceed 1000. Hence, based on the IUCN criteria (IUCN 2024), *V.liupanshanensis* is here classified as Vulnerable (VU).

### ﻿Key to Chinese Species of Section Cassubicae

**Table:** 

1a	Stipules large, over 10 mm long	**2**
2a	Leaflets small, 1.3–4 cm long, 0.5–1.8 cm wide; leathery; apex rounded or slightly emarginate, lateral veins fan-shaped, extending to margin without forming reticulation	**1. *V.amoena***
2b	Leaflets large, (2-)3–6(-10) cm long, 1.2–2.5 cm wide; papery; apex acute, lateral veins reaching margin in wavy or dentate connections	**2. *V.pseudo-orobus***
1b	Stipules small, less than 10 mm long	**3**
3a	Flowers purple, bluish-purple or red	**4**
4a	Flowers small, 0.7–1.4 cm long	**3. *V.kioshanica***
4b	Flowers larger, 10–20 mm long	**5**
5a	Leaflet venation dense and distinct, lateral veins spreading at right angles	**6**
6a	Leaflets elliptic or oblong-ovate, 10–16 mm wide; flowers 15–30, densely arranged	**4. *V.amurensis***
6b	Leaflets oblong, less than 6 mm wide; flowers 4–15, loosely arranged	**7**
7a	Stipules small, bifid; inflorescence nearly equaling leaves; flowers 10–11 mm long	**5. *V.perelegans***
7b	Stipules triangular, with several teeth; inflorescence longer than leaves; flowers 10–20 mm long	**6. *V.tibetica***
5b	Leaflet venation sparse, lateral veins ascending at acute angles	**8**
8a	Flowers with bracteoles; inflorescence shorter than leaves; stipules semi-sagittate, rhombic to lanceolate; leaflets 6–10 pairs, linear-lanceolate, ovate-lanceolate or oblong	**7. *V.latibracteolata***
8b	Flowers without bracteoles; inflorescence equaling or slightly exceeding leaves; stipules bifid, lobes subulate	**9**
9a	Leaflets linear-oblong, very narrow, only 1.5–3 mm wide; inflorescence longer than leaves; flowers 13–17 mm long	**8. *V.multicaulis***
9b	Leaflets elliptic or ovate-lanceolate, 10–20 mm long, relatively broad; inflorescence equaling leaves; flowers 10–14 mm long	**10**
10a	Leaflets ovate-lanceolate, 4–7 mm wide	**9. *V.chinensis***
10b	Leaflets elliptic, broadly elliptic to long-ovate, 6–14 mm wide	**10. *V.japonica***
3b	Flowers dull yellow or white	**11**
11a	Plants tall, shrubby, white-pubescent; flowers smallca. 7 mm long	**11. *V.sinogigantea***
11b	Plants sparsely pubescent or glabrous; flowers relatively large	**12**
12a	Leaflets large, 15–49 mm long, 6–15 mm wide	**13**
13a	Flowers white; inflorescence usually branched, shorter than leaves	**12. *V.wushanica***
13b	Flowers dull yellow or pale blue; inflorescence nearly equaling or longer than leaves	**14**
14a	Stipules entire; inflorescence axis slender	**15**
15a	Leaflets 4–6 pairs; inflorescence without bracteoles	**13. *V.taipaica***
15b	Leaflets 7–9 pairs; inflorescence with bracteoles	**14. *V.liupanshanensis***
14b	Stipules divided; inflorescence axis thick and straight	**16**
16a	Flowers dull yellow; leaflets gray-green beneath; 20–25 flowers	**15. *V.dichroantha***
16b	Flowers blue-yellow; leaflets gray-green beneath; 15–20 flowers	**16. *V.ternata***
12b	Leaflets small, 6–15 mm long, 2–5 mm wide	**17**
17a	Flowers white or pale yellow; inflorescence longer than leaves, with 3–11 flowers; leaflets oblong-lanceolate or elliptic; occurring in Xinjiang	**17. *V.costata***
17b	Flowers yellow; inflorescence equaling or slightly exceeding leaves, with 6–9(-12) flowers; leaflets elliptic; occurring in Southwest China	**18. *V.nummularia***

## ﻿Discussion

*Vicialiupanshanensis* belongs to Subgen. Cracca due to its perennial herbaceous and climbing habit, as well as the presence of tendrils. *Vicialiupanshanensis* is characterized by the following morphological features: leaves that are approximately 2.5 times longer than wide; inflorescences that bear around 15 flowers; leaflets that are elliptic, ovate or lanceolate in shape, with typically conspicuous venation patterns; and a perennial growth habit. Based on these diagnostic characteristics, it should be classified within Section Cassubicae. Upon reviewing the data, we discovered that specimens of *V.liupanshanensis* were previously misidentified as *V.costata*. These two species differ distinctly in habitat and morphology. Morphologically, *V.costata* has leaves with 3–8 pairs of leaflets, racemes longer than their leaves, and yellow to white flowers with bluish-purple veins. In contrast, *V.liupanshanensis* is distinguished by having leaves with 7–9 pairs of leaflets, racemes that are the same length as their subtending leaves, and pale greenish-creamy-white flowers that turn yellowish-brown to dark orange; these lacking any bluish-purple veins. Additionally, *Viciacostata* prefers arid deserts, gravelly slopes, and sandy beaches, whereas *V.liupanshanensis* is found in alpine scrubs or grassy slopes. In comparison to *V.costata*, both *V.taipaica* and *V.mingyueshanensis* are found in relatively humid habitats, such as forest edges and the forest understorey. Additionally, the colour of their flowers changes from pale to dark yellow, and they consistently grow to a length of approximately 1 cm.

Located in northwestern China, the Liupan Mountains have a temperate, semi-humid semi-arid monsoon climate, creating a rather unique ecological environment. This area is rich in biodiversity, including 1347 identified species of wild vascular plants and 384 species of wild vertebrates. This is why it is known as the “gene pool of germplasm resources in northwestern China” (Bao et al. 2023). The discovery of *V.liupanshanensis* is particularly significant, as it not only enriches the number of known *Vicia* species, but it could also be instrumental in bolstering the entire flora of the Liupan Mountains. Investigating the key evolutionary characteristics of *V.liupanshanensis* in comparison to other *Vicia* species could help to clarify the phylogenetic relationships within this genus. These findings will provide crucial evidence for a more detailed analysis of the complex ecological interactions among leguminous plants in this region. Such knowledge can also be used for improving their *in situ* conservation in general, and that of *V.liupanshanensis* in particular.

## Supplementary Material

XML Treatment for Vicia liupanshanensis

## ﻿Additional information

### Author ORCIDs

Bo Yang https://orcid.org/0000-0002-7812-4375

Jun Yang https://orcid.org/0000-0002-5037-484X

Siyu Wei https://orcid.org/0009-0009-5212-8966

Hongmei Zhang https://orcid.org/0000-0002-2691-3655

Xiaowei Li https://orcid.org/0009-0004-2060-7384

### Data availability

All of the data that support the findings of this study are available in the main text.
